# Supplementary material for: Stress responses to conspecific visual cues of predation risk in zebrafish
Source: PeerJ. 2017 Sep 4;5:e3739. doi: 10.7717/peerj.3739 (PMC5588784; doi:10.7717/peerj.3739)
Supplement: Supplemental Information 1 — Whole body cortisol and behavioral analysis data and statistics. [file peerj-05-3739-s001.pdf]

(Data and statistics)

Title: Stress responses to conspecific visual cues of predation risk in zebrafish

Authors: Thiago Acosta Oliveira; Renan Idalencio, Fabiana Kalichack, João Gabriel S. da Rosa, Gessi Koakoski, Murilo S. de Abreu, Ana Cristina V. Giacomini, Darlan Gusso, Denis B. Rosemberg, Rodrigo E. Barreto, Leonardo José Gil Barcellos

Whole-body cortisol raw data expressed as ng/g of wet tissue

Predator		Non-predator		Non stimulus fish	
Sender	Receiver	Sender	Receiver	Sender	Receiver
47.5	20.0	10.5	5.5	5.0	9.0
15.0	14.5	11.5	9.5	8.5	18.0
23.5	14.0	24.0	8.5	9.0	14.5
17.0	22.5	28.0	9.5	9.0	9.0
19.5	17.0	22.5	9.5	10.0	12.0
17.0	16.5	14.5	6.0	6.0	2.5
17.5	19.0	16.5	7.0	4.5	13.0
	15.0		7.5	8.5	

Whole-body cortisol statistics

Two-way ANOVA	Ordinary				
Alpha	0,05				
Source of Variation	% of total variation	P value	P value summary	Significant?	
Interaction	13,67	0,0077	**	Yes	
Row Factor	6,499	0,0274	*	Yes	
Column Factor	31,50	< 0,0001	****	Yes	
ANOVA table	SS	DF	MS	F (DFn, DFd)	P value
Interaction	369,2	2	184,6	F (2, 39) = 5,527	P = 0,0077
Row Factor	175,5	1	175,5	F (1, 39) = 5,254	P = 0,0274
Column Factor	850,4	2	425,2	F (2, 39) = 12,73	P < 0,0001
Residual	1303	39	33,40		

Number of families	1
Number of comparisons per family	15
Alpha	0,05

Tukey's multiple comparisons test	Mean Diff,	95% CI of diff,	Significant?	Summary
Sender:Predator vs. Sender:Non-predator	4,214	-5,041 to 13,47	No	ns
Sender:Predator vs. Sender:Non-stimulus fish	14,87	5,905 to 23,83	Yes	***
Sender:Predator vs. Receiver:Predator	5,116	-3,845 to 14,08	No	ns
Sender:Predator vs. Receiver:Non-predator	14,55	5,593 to 23,51	Yes	***
Sender:Predator vs. Receiver:Non-stimulus fish	11,29	2,031 to 20,54	Yes	**
Sender:Non-predator vs. Sender:Non-stimulus fish	10,65	1,691 to 19,61	Yes	*
Sender:Non-predator vs. Receiver:Predator	0,9018	-8,059 to 9,863	No	ns
Sender:Non-predator vs. Receiver:Non-predator	10,34	1,378 to 19,30	Yes	*
Sender:Non-predator vs. Receiver:Non-stimulus fish	7,071	-2,183 to 16,33	No	ns
Sender:Non-stimulus fish vs. Receiver:Predator	-9,750	-18,41 to -1,093	Yes	*
Sender:Non-stimulus fish vs. Receiver:Non-predator	-0,3125	-8,970 to 8,345	No	ns
Sender:Non-stimulus fish vs. Receiver:Non-stimulus fish	-3,580	-12,54 to 5,381	No	ns
Receiver:Predator vs. Receiver:Non-predator	9,438	0,7803 to 18,09	Yes	*
Receiver:Predator vs. Receiver:Non-stimulus fish	6,170	-2,791 to 15,13	No	ns
Receiver:Non-predator vs. Receiver:Non-stimulus fish	-3,268	-12,23 to 5,693	No	ns

Sender fish

Table Analyzed	senders
Kruskal-Wallis test	
P value	0,0006
Exact or approximate P value?	Approximate
P value summary	***
Do the medians vary signif. (P < 0.05)	Yes
Number of groups	3
Kruskal-Wallis statistic	14,92
Data summary	
Number of treatments (columns)	3
Number of values (total)	22

Post-hoc

Number of families	1				
Number of comparisons per family	3				
Alpha	0,05				
Dunn's multiple comparisons test	Mean rank diff,	Significant?	Summary		
predator SF vs. non-predator SF	1,857	No	ns		
predator SF vs. non-stimulus fish SF	11,93	Yes	**		
non-predator SF vs. non-stimulus fish SF	10,07	Yes	**		
Test details	Mean rank 1	Mean rank 2	Mean rank diff,	n1	n2
predator SF vs. non-predator SF	16,43	14,57	1,857	7	7
predator SF vs. non-stimulus fish SF	16,43	4,500	11,93	7	8
non-predator SF vs. non-stimulus fish SF	14,57	4,500	10,07	7	8

Receiver fish

Table Analyzed	receivers
Kruskal-Wallis test	
P value	0,0010
Exact or approximate P value?	Approximate
P value summary	**
Do the medians vary signif. (P < 0.05)	Yes
Number of groups	3
Kruskal-Wallis statistic	13,74
Data summary	
Number of treatments (columns)	3
Number of values (total)	23

Post-hoc

Number of families	1				
Number of comparisons per family	3				
Alpha	0,05				
Dunn's multiple comparisons test	Mean rank diff,	Significant?	Summary		
predator RF vs. non-predator RF	12,44	Yes	***		
predator RF vs. non-stimulus fish RF	7,759	No	ns		
non-predator RF vs. non-stimulus fish RF	-4,679	No	ns		
Test details	Mean rank 1	Mean rank 2	Mean rank diff,	n1	n2
predator RF vs. non-predator RF	18,69	6,250	12,44	8	8
predator RF vs. non-stimulus fish RF	18,69	10,93	7,759	8	7
non-predator RF vs. non-stimulus fish RF	6,250	10,93	-4,679	8	7

Behavior – expressed as % of time spent at the tank bottom

Predator		Non-predator		Non stimulus fish	
Sender	Receiver	Sender	Receiver	Sender	Receiver
92,77778	71,38889	63,88889	63,61111	8,611111	8,888889
93,33333	100,00000	36,11111	34,72222	12,222220	16,111110
96,38889	100,00000	58,88889	50,00000	16,388890	11,111110
86,38889	93,05556	77,77778	43,05556	23,333330	23,611110

Statistics

Two-way ANOVA	Ordinary				
Alpha	0,05				
Source of Variation	% of total variation	P value	P value summary	Significant?	
Interaction	0,5870	0,5474	ns	No	
Row Factor	0,4104	0,3629	ns	No	
Column Factor	90,52	< 0,0001	****	Yes	
ANOVA table	SS	DF	MS	F (DFn, DFd)	P value
Interaction	152,3	2	76,16	F (2, 18) = 0,6231	P = 0,5474
Row Factor	106,5	1	106,5	F (1, 18) = 0,8713	P = 0,3629
Column Factor	23490	2	11745	F (2, 18) = 96,09	P < 0,0001
Residual	2200	18	122,2		

Post hoc

Compare cell means regardless of rows and columns	
Number of families	1
Number of comparisons per family	15
Alpha	0,05

Tukey's multiple comparisons test	Mean Diff,	95% CI of diff,	Significant?	Summary
Sender:predator vs. Sender:non-predator	33,06	8,211 to 57,90	Yes	**
Sender:predator vs. Sender:non-stimulus fish	77,08	52,24 to 101,9	Yes	****
Sender:predator vs. Receiver:predator	1,111	-23,73 to 25,96	No	ns
Sender:predator vs. Receiver:non-predator	44,38	19,53 to 69,22	Yes	***
Sender:predator vs. Receiver:non-stimulus fish	77,29	52,45 to 102,1	Yes	****
Sender:non-predator vs. Sender:non-stimulus fish	44,03	19,18 to 68,87	Yes	***
Sender:non-predator vs. Receiver:predator	-31,94	-56,79 to -7,100	Yes	**
Sender:non-predator vs. Receiver:non-predator	11,32	-13,52 to 36,16	No	ns
Sender:non-predator vs. Receiver:non-stimulus fish	44,24	19,39 to 69,08	Yes	***
Sender:non-stimulus fish vs. Receiver:predator	-75,97	-100,8 to -51,13	Yes	****
Sender:non-stimulus fish vs. Receiver:non-predator	-32,71	-57,55 to -7,864	Yes	**
Sender:non-stimulus fish vs. Receiver:non-stimulus fish	0,2083	-24,64 to 25,05	No	ns
Receiver:predator vs. Receiver:non-predator	43,26	18,42 to 68,11	Yes	***
Receiver:predator vs. Receiver:non-stimulus fish	76,18	51,34 to 101,0	Yes	****
Receiver:non-predator vs. Receiver:non-stimulus fish	32,92	8,073 to 57,76	Yes	**

Sender fish

Table Analyzed	senders beahv				
ANOVA summary					
F	50,17				
P value	< 0,0001				
P value summary	****				
Are differences among means statistically significant? (P < 0.05)	Yes				
R square	0,9177				
Brown-Forsythe test					
F (DFn, DFd)	1,841 (2, 9)				
P value	0,2136				
P value summary	ns				

Significantly different standard deviations? (P < 0.05)	No				
Bartlett's test					
Bartlett's statistic (corrected)	5,438				
P value	0,0659				
P value summary	ns				
Significantly different standard deviations? (P < 0.05)	No				
ANOVA table	SS	DF	MS	F (DFn, DFd)	P value
Treatment (between columns)	11964	2	5982	F (2, 9) = 50,17	P < 0,0001
Residual (within columns)	1073	9	119,2		
Total	13037	11			
Data summary					
Number of treatments (columns)	3				
Number of values (total)	12				

Post hoc

Number of families	1							
Number of comparisons per family	3							
Alpha	0,05							
Tukey's multiple comparisons test	Mean Diff,	95% CI of diff,	Significant?	Summary				
predator SF vs. non-predator SF	33,06	11,50 to 54,61	Yes	**				
predator SF vs. non-stimulus fish SF	77,08	55,53 to 98,64	Yes	****				
non-predator SF vs. non-stimulus fish SF	44,03	22,47 to 65,59	Yes	***				
Test details	Mean 1	Mean 2	Mean Diff,	SE of diff,	n1	n2	q	DF
predator SF vs. non-	92,22	59,17	33,06	7,721	4	4	6,055	9

predator SF								
predator SF vs. non-stimulus fish SF	92,22	15,14	77,08	7,721	4	4	14,12	9
non-predator SF vs. non-stimulus fish SF	59,17	15,14	44,03	7,721	4	4	8,064	9

Receiver fish

Table Analyzed	receivers behav				
ANOVA summary					
F	46,63				
P value	< 0,0001				
P value summary	****				
Are differences among means statistically significant? (P < 0.05)	Yes				
R square	0,9120				
Brown-Forsythe test					
F (DFn, DFd)	0,3653 (2, 9)				
P value	0,7039				
P value summary	ns				
Significantly different standard deviations? (P < 0.05)	No				
Bartlett's test					
Bartlett's statistic (corrected)	1,354				
P value	0,5080				
P value summary	ns				
Significantly different standard deviations? (P < 0.05)	No				
ANOVA table	SS	DF	MS	F (DFn, DFd)	P value
Treatment (between columns)	11678	2	5839	F (2, 9) = 46,63	P < 0,0001
Residual (within columns)	1127	9	125,2		
Total	12805	11			

Data summary					
Number of treatments (columns)	3				
Number of values (total)	12				

Post hoc

Number of families	1							
Number of comparisons per family	3							
Alpha	0,05							
Tukey's multiple comparisons test	Mean Diff,	95% CI of diff,	Significant?	Summary				
predator RF vs. non-predator RF	43,26	21,17 to 65,36	Yes	**				
predator RF vs. non-stimulus fish RF	76,18	54,09 to 98,27	Yes	****				
non-predator RF vs. non-stimulus fish RF	32,92	10,82 to 55,01	Yes	**				
Test details	Mean 1	Mean 2	Mean Diff,	SE of diff,	n1	n2	q	DF
predator RF vs. non-predator RF	91,11	47,85	43,26	7,913	4	4	7,733	9
predator RF vs. non-stimulus fish RF	91,11	14,93	76,18	7,913	4	4	13,62	9
non-predator RF vs. non-stimulus fish RF	47,85	14,93	32,92	7,913	4	4	5,883	9
